# Impact of proton therapy on antitumor immune response

**DOI:** 10.1038/s41598-021-92942-1

**Published:** 2021-06-29

**Authors:** Céline Mirjolet, Anaïs Nicol, Emeric Limagne, Carole Mura, Corentin Richard, Véronique Morgand, Marc Rousseau, Romain Boidot, François Ghiringhelli, Georges Noel, Hélène Burckel

**Affiliations:** 1 Department of Radiation Oncology, Unicancer - Georges-Francois Leclerc Cancer Center, 21000 Dijon, France; 2 INSERM UMR 1231, 21000 Dijon, France; 3 Radiobiology Laboratory, Paul Strauss Comprehensive Cancer Center, Institut de Cancérologie Strasbourg Europe (ICANS), Strasbourg University, UNICANCER, 67000 Strasbourg, France; 4 Cancer Biology Research Platform, Unicancer - Georges-Francois Leclerc Cancer Center, 21000 Dijon, France; 5 CNRS, IPHC, UMR 7178, Strasbourg University, 67200 Strasbourg, France; 6 Molecular Biology Clinical Research, Unicancer - Center Georges-Francois Leclerc, 21000 Dijon, France; 7 Department of Radiation Oncology, Paul Strauss Comprehensive Cancer Center, Institut de Cancérologie Strasbourg Europe (ICANS), UNICANCER, 17 Rue Albert Calmette, 67200 Strasbourg, France

**Keywords:** Cancer microenvironment, Cancer therapy, Tumour immunology, Cancer, Oncology, Cancer therapy, Radiotherapy

## Abstract

Radiotherapy delivered using photons induces an immune response that leads to modulation of the tumor microenvironment. Clinical studies are ongoing to evaluate immune checkpoint inhibitors in association with photon radiotherapy. At present, there is no publication on the radio-induced immune response after proton therapy. Balb/c mice bearing subcutaneous CT26 colon tumors were irradiated by a single fraction of 16.4 Gy using a proton beam extracted from a TR24 cyclotron. RNA sequencing analysis was assessed at 3 days post-treatment. Proton therapy immune response was monitored by flow cytometry using several panels (lymphoid, myeloid cells, lymphoid cytokines) at 7 and 14 days post-irradiation. RNA-Seq functional profiling identified a large number of GO categories linked to “immune response” and “interferon signaling”. Immunomonitoring evaluation showed induced tumor infiltration by immune cells. This is the first study showing the effect of proton therapy on immune response. These interesting results provide a sound basis to assess the efficacy of a combination of proton therapy and immune checkpoint inhibitors.

## Introduction

Radiation therapy is used in more than 50% of cancer patients. Initially, radiotherapy (RT) was used for its direct effects on cancer cell survival. In addition to its cytotoxic action on cancer cells, it is now well established that irradiation also induces an immune response^1^. This can be immune-activating and/or immunosuppressive^2^. These immune mechanisms can be observed in the tumor microenvironment (TME). Briefly, optimized RT delivered on tumor can induce an immunogenic cell death (ICD) characterized by a release of cytokines and damage associated molecular patterns (DAMPs). These signals induced the recruitment of antigen presenting cells (APC), such as dendritic cells (DCs), inducing the processing of tumor associated antigens (TAAs) and cross presentation of antigenic peptides on major histocompatibility complex class I (MHC I), whose expression is radio-induced^3–5^. Cross presentation of tumor antigens can lead to activation of tumor-specific T lymphocytes and theirs trafficking into tumors. Another type of activation and the production of type-I interferon (IFN) can also occur, increasing activation of both DCs and T cells. Furthermore, RT induced DNA damages, whose fragments leak to cytosolic compartment of cells. These cytosolic DNA fragments can activate the cGAS-STING-IRF3-type I IFN-mediated DCs recruitment for cross presentation and immune response^6–8^.

Although, in contrast to these immune-activation effects, RT can also lead to immune-suppressive responses by inducing regulatory T lymphocytes (Tregs) tumor-infiltration, or by promoting immunosuppressive immune effector cells, such as macrophages and other myeloid derived cells^2^.

For the past ten years, the association of radiotherapy and immunotherapy in preclinical, and more recently in clinical settings, has been in full development and seems promising for many indications. In order to improve the effectiveness of these combinations, it is necessary to optimize the modalities of radiotherapy administration, such as the dose^9^, the fractionation scheme^10^ or the dose rate^11^. Almost all the data in the literature regarding radio-induced immune response were obtained with irradiation based on photons.

It is becoming essential to evaluate another feature of radiotherapy, namely the impact of the type of particles used. Proton and carbon ion irradiations of tumors were also described to induce an immune response. Most of these studies evaluated immune-response related components on tumor cells in vitro, rather than direct stimulation of specific antitumor immunity in vivo. The expression of some ICD markers, such as calreticulin and MHC-class 1, has been analyzed following proton radiation. Proton radiation of several types of cancer cells upregulated the expression of these markers of immunogenic modulation, with degrees of upregulation similar to those observed after equivalent exposure to photon radiation^12,13^. Recently, Spina et al*.*, evaluated the effects of carbon-ion therapy on immune modulation. They highlighted an interesting induction of pro-inflammatory cytokines^14^. Yoshimoto et al. evaluated the immunogenic alterations induced by carbon ion irradiation in vitro. They described that carbon ion radiation increased the secretion of high mobility group box 1 (HMGB1) in human cancer cell lines. HMGB1 is an ICD marker, playing an important role in antigen-presenting cell activation and induction of immune response^15^. The levels of HMGB1 induced by carbon ion exposure were similar with equivalent doses of photon irradiation. Although the number of patients treated with protons is clearly higher than with carbon ion, preclinical studies with particles have focused on the impact of carbon ion irradiation on immune response. Preclinical in vivo studies using carbon ion radiation have convincingly demonstrated that this type of particle induces antitumor immune response in immunocompetent animals^16^.

Proton therapy (PT) plays an important role in clinical radiotherapy with growing facilities and indications^17^. It has particularly interesting ballistic advantages defined by the Bragg peak, beyond which the dose delivered is almost nil, enabling the total avoidance of surrounding organs at risk. Protons have an increased Relative Biological Effectiveness (RBE) compared to photons, and they cause a different type of damage to DNA than do photons^18^. Moreover, densely ionizing radiation may have other biological advantages induced by different cell death pathways and release of pro-inflammatory cytokines^19^.

Recently, Durante and Formenti proposed that, combined with immunotherapy, particle radiation could be more effective than photon radiation, as protons and heavy ions displayed physical advantages and led to reduced damage to blood lymphocytes that are required for an effective anti-tumor immune response^20^. However, the effect of proton on the intratumor immune response remains currently unexplored.

In this study, we aimed to evaluate the radio-induced immune response with a 16.4 Gy single fraction of proton. We evaluated this response by transcriptomic analysis in order to describe the immune molecular pathways modulated by proton therapy, and by analysis of the tumor microenvironment, by immunomonitoring of intratumoral infiltrated immune cells.

## Results

### Effect of 16.4 Gy with proton therapy on CT26 tumor growth

**Figure 1:**
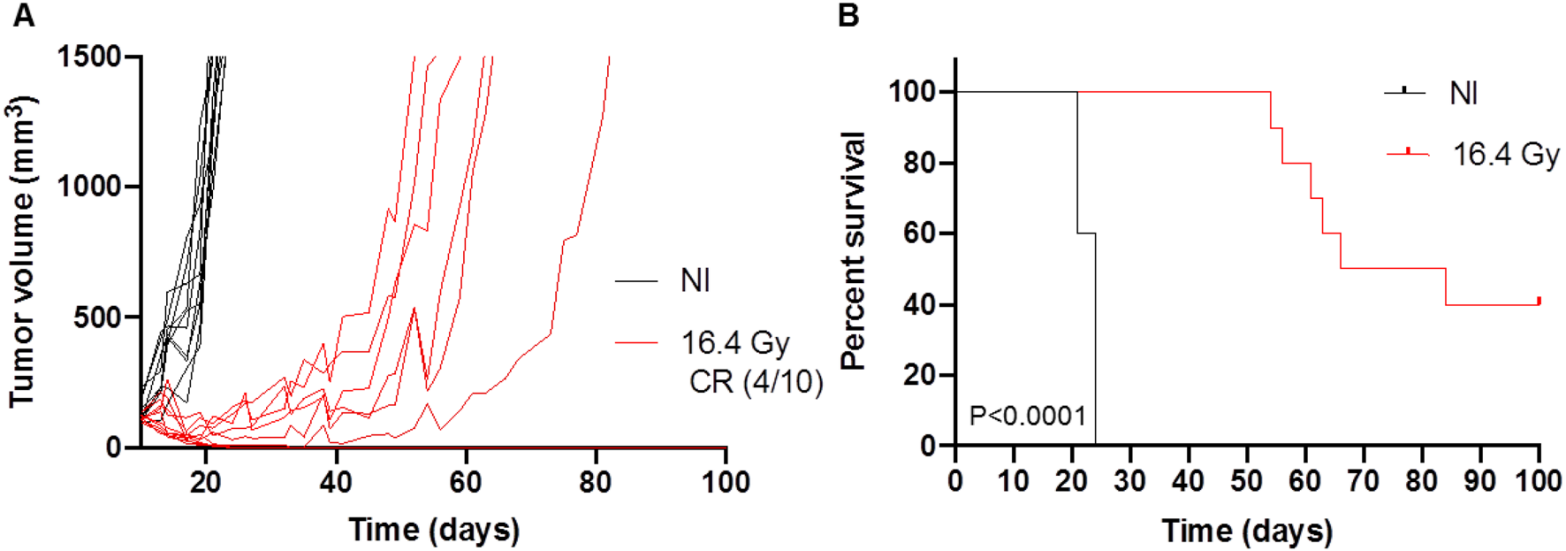
Effect of a single dose 16.4 Gy proton beam irradiation on CT26 tumors volumes implanted on immunocompetent BALB/c mice (red) compared to non-irradiated (NI) tumors (black) (**A**) and Kaplan Meier survival curves with log-rank test comparison (**B**). n = 10 mice per group. CR: complete response. Ten days after injection of CT26 colon murine cancer cells into Balb/c mice, tumors were irradiated.

A single dose of 16.4 Gy proton therapy was delivered to the CT26 tumors of immunocompetent BALB/c mice. The dose of 16.4 Gy delivered with proton therapy induced enhanced CT26 tumor control compared to the non-irradiated (NI) control tumors (Fig. 1A,B). We highlighted a significant tumor growth delay for the proton therapy group, compared to NI control (*P* < 0.0001), with median survivals of 75 versus 24 days, respectively. For 6 mice out of 10 treated by 16.4 Gy proton therapy, the maximum tumor volume limit of 1500 mm^3^ was reached between 54 and 84 days, while the 4 remaining mice still presented complete response after 100 days.

### Proton therapy activated transcriptomic antitumor immune response pathways

Gene transcript analysis was compared between 16.4 Gy and non-irradiated CT26 tumors, 3 days post-irradiation to examine immune response after proton therapy. RNA-Seq profiling identified 68 genes differentially expressed with s-value < 0.005 and a Fold Change ≥ 2 (Table S1).

Enrichment analysis was then performed on the gene set (Table S2), then we focused on pathways linked to “immune response” and “interferon signaling”. The enriched GO categories listed in Table 1 represent all the pathways identified that included “immune response” and “interferon signaling”.

**Table 1:** List of GO categories obtained after enrichment on gProfiler2 and linked to “immune response” and “interferon signaling” after a dose of 16.4 Gy proton therapy on CT26 tumors with an s-value < 0.005 and a Fold Change ≥ 2.

Id	Term_id	Term_name	Intersection_size	*P*_value
1	GO:0045087	Innate immune response	21	8.2e−11
2	GO:0002252	Immune effector process	19	5.1e−09
3	GO:0002376	Immune system process	31	7.0e−09
4	GO:0006955	Immune response	25	9.6e−09
5	GO:0034097	Respose to cytokine	18	3.2e−07
6	GO:0071345	Cellular response to cytokine stimulus	15	3.0e−05
7	GO:0035456	Response to interferon-beta	6	5.4e−05
8	GO:0035457	Cellular response to interferon-alpha	4	1.8e−04
9	GO:0035458	Cellular response to interferon-beta	5	7.0e−04
10	GO:0002682	Regulation of immune system process	17	7.9e−04
11	GO:0035455	Response to interferon-alpha	4	3.4e−03
12	GO:0032728	Positive regulation of interferon-beta production	4	5.1e−03
13	GO:0002218	Activation of initiate immune response	4	1.2e−02
14	GO:0032608	Interferon-beta production	4	3.0e−02
15	GO:0045088	Regulation of initiate immune response	6	3.4e−02
16	GO:0045089	Positive regulation of initiate immune response	5	3.7e−02

**Figure 2:**
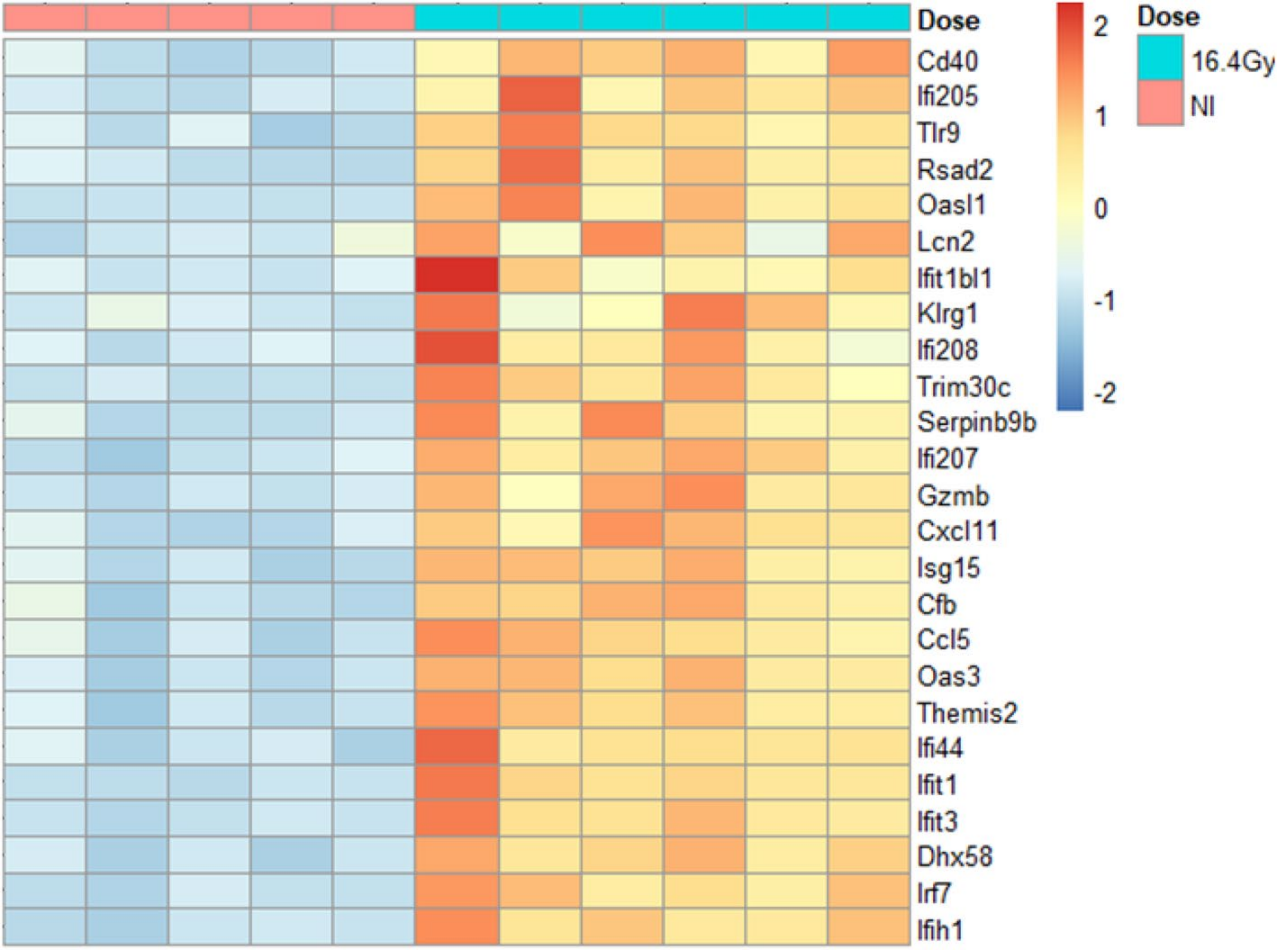
Heatmap representing “immune response” biological process, with 25 genes differentially expressed, at 3 days post-irradiation. Comparison between 16.4 Gy (green) and non-irradiated controls (NI, pink) of CT26 tumors. RNA-Seq profiling analysis was assessed with R software (R version 4.0.3) according to guidelines^21^. s-value < 0.005 and a Fold Change ≥ 2. Groups contained 5 mice for NI and 6 mice for 16.4 Gy.

Next, we extracted all genes involved in the “immune response” pathway obtained with the enrichment (*p* value = 9.6e−09). This pathway was covered by 25 differentially expressed genes, which are presented in a heatmap below (Fig. 2). All the genes observed presented up-regulation after 16.4 Gy proton therapy, compared to NI controls. Among these genes, we identified several genes of *Ifit* (Interferon-induced protein with tetratricopeptid repeats) and *Ifi* (Interferon inducible protein) families, which are involved in Interferon alpha and beta signaling pathways. Many induced genes are involved in the type I interferon pathway.

**Figure 3:**
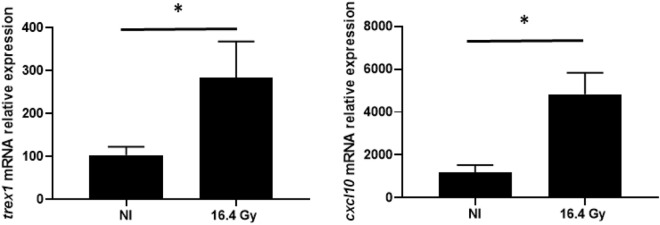
Quantification of *Cxcl10* and *Trex1* genes mRNA relative expression, using comparative ΔCt method. Data represent the mean ± SD (n = 5 for NI and 6 for 16.4 Gy). The non-parametric Mann–Whitney test was used.*: *P* < 0.05.

Therefore, we looked up for the expression of genes of interest relative to Interferon Type 1 pathway in our RNASeq profiling, *Ifnar2*, *Cxcl10* and *Trex1* tended to be over-expressed after 16.4 Gy of proton therapy, but with s-values > 0.005 (data not shown). As *Cxcl10* and *Trex1* presented Fold Change > 2, we decided to quantify their variation of expression using RTqPCR. Both *Cxcl10* and *Trex1* displayed a significant increase in their relative expression (*p* < 0.05), validating the results obtained by RNAseq analysis on those genes (Fig. 3).

### Proton therapy induced intra-tumor immune cell infiltration

**Figure 4:**
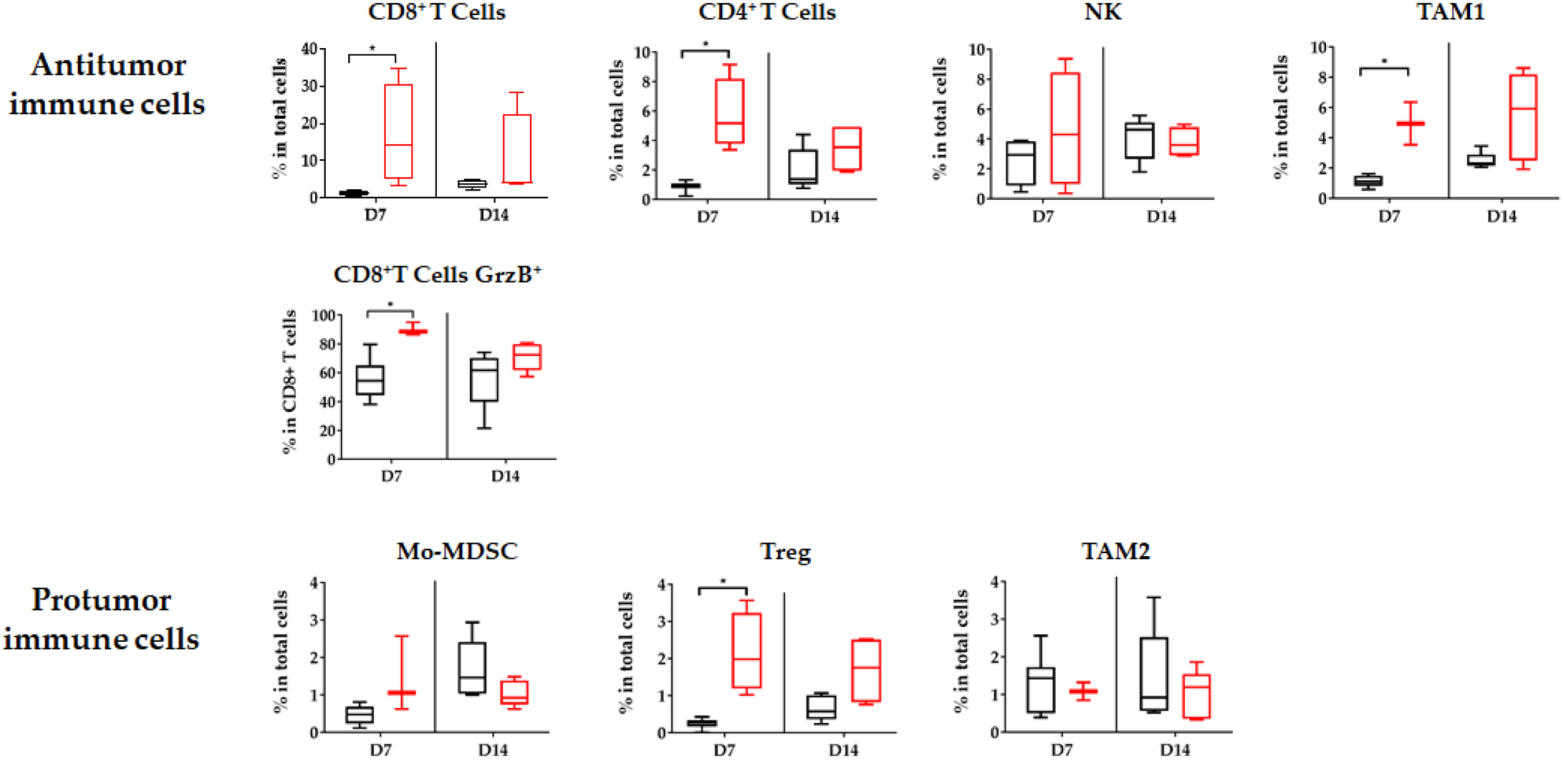
Modification of the tumor microenvironment induced by proton therapy: immunomonitoring of lymphoid and myeloid cells after proton therapy. At 7 and 14 days after PT, flow cytometry monitoring (FCM) was performed on dissociated tumors. Antitumor immune cell (CD8^+^ T cells, CD4^+^ T cells, Natural Killer (NK) cells, tumor associated macrophages (TAM) 1 and CD8^+^ Granzyme B^+^ (GrzB) and pro-tumor cell (myeloid derived suppressor cells (Mo-MDSC), Treg T cells and TAM2) infiltration was quantified. Black: non-irradiated control, Red: 16.4 Gy PT. All data were expressed in percentages of total cells, except for CD8^+^T cells Granzyme B^+^ which were expressed in percentages of CD8 + T cells. All data are shown with box and whisker plots, with min to max values obtained from 4–5 independent samples per point. The results are expressed as mean ± SEM. The non-parametric Mann–Whitney test was used. *p < 0.05.

Proton therapy induced infiltration of two types of immune cells: antitumor and pro-tumor immune cells (Fig. 4). Seven days after PT, significant infiltration of CD8^+^ T cells, CD4^+^ T cells and type 1 tumor associated-macrophage (TAM1) was observed. For these cells, infiltration tended to be maintained 14 days after PT, albeit without reaching statistical significance. We did not observe radio-induced infiltration of NK cells in these conditions. CD8^+^ T cells presented a cytotoxic activity phenotype, with co-expression of Granzyme B in more than 80% of them. Concerning immunosuppressive cells, we observed significant tumor infiltration of Treg, but no significant variation in myeloid derived suppressor cells (Mo-MDCS) and type 2 tumor associated-macrophage (TAM2) infiltrations, 7 and 14 days after PT (Fig. 4). All significant infiltrations were transient, with loss of significance of these infiltrates compared to controls at D14.

## Discussion

Proton therapy is an attractive therapeutic approach. It has both physical advantages, limiting the dose to surrounding organs at risk, and biological advantages, due to high linear energy transfer with different types of DNA damage induced by the densely ionizing radiation^22^. Several teams have described the potential effects of protons on the antitumor immune response, illustrating the importance of this issue, but also the difficulty of implementing experiments to demonstrate it^16,20,23,24^. Devices amenable to carrying out such studies in vivo are rare, and offer limited access for research studies. For example, in France, only few machines are available, and only a few slots per month are opened for research.

A 16.4 Gy unique dose of proton therapy significantly enhanced the time to reach the tumor volume limit, with 4 mice out of 10 with complete response 100 days post-irradiation. This model was never used with proton therapy. Nevertheless, the data obtained by Grapin et al. on the same colon carcinoma tumor model, after 16.4 Gy X-rays, presented increased growth delays without any complete response compared to our results^10^. In addition, this single dose of photon irradiation was compared to two fractionation schedules, with the same Biologically Effective Dose (BED), 3 × 8 Gy and 18 × 2 Gy (Figure S1). Whatever the fractionation with photon irradiation, high single dose, hypofractionation or “classical” fractionation, our single dose of proton therapy increased more tumor growth delay.

This present study highlights that proton therapy enables the activation of several pathways implicated in the immune response. Activation of innate and specific immune responses was previously described to be induced by type I interferon (IFN-I), with activation of antigen recognition and cytolytic activity^25,26^. One of the critical signals for the spontaneous activation of antitumor T cells against immunogenic tumors, as well as RT-induced T-cell priming, is double strand DNA (dsDNA) quantity^27^. Cytosolic DNA derived dsDNA is sensed by cyclic GMP-AMP synthase (cGAS) to generate cGAMP required for the activation of the adaptor STING (stimulator of interferon genes), resulting in the induction of several interferon-stimulated genes such as Cxcl10^8,9,26,28–30^. Cxcl10 served as a chemotactic gradient to recruit T cells, expressing CXCR3, inside tumor^31^.

After conventional RT, several papers demonstrated that photon RT induced cytosolic DNA accumulation, which activates the cGAS/STING pathway resulting in activation of IFN-I^9,32,33^. In this same publication, authors also reported that doses at 8 and 10 Gy per fraction led to Trex1 expression, which degrades cytosolic DNA, thus avoiding IFN-I activation^2^.

Harding et al. showed that radio-induced activation of type I IFN signalling was preceded by micronuclei formation^34^. The formation of micronuclei is one of the mechanisms responsible for the presence of cytosolic DNA. DNA double-strand breaks are the most lethal lesions induced by ionizing radiation and can trigger a series of cellular DNA damage responses (DDRs). Recently, a review described the current evidence linking the DNA damage responses to activation of the immune response through micronuclei formation^35^. It has been demonstrated that proton therapy induced more DNA double-strand breaks and micronuclei than photon irradiation at equivalent doses (2–12 Gy)^36^. The IFN-I pathway was induced with a single dose of 16.4 Gy proton therapy in our study (ie. up-regulation of Ifi and Ifit genes in RNASeq). These results suggest that the proton-induced mechanisms could be comparable to those induced by conventional photon radiotherapy.

With a high dose per fraction of photon, Vanpouille-Box et al. described an increase in Trex1 expression as well as a decrease in the amount of cytosolic DNA and a decrease in the activation of cGAS/STING in TSA cells, highlighted by a Cxcl10 expression decrease^9^ (supplementary data). With our gene transcript analysis, we also observed an increasing expression of Trex1 in our group irradiated with 16.4 Gy proton therapy. However, we observed Cxcl10 gene expression induction. Other authors have previously described an induction of Cxcl10 expression after hypofractionated photon therapy administrated by 2 × 12 Gy using B16 cancer model^37^. It would be interesting to also evaluate in vitro the effect of protons on the amount of cytosolic DNA.

As described in the review of Zhang et al.^8^, two DNA sensing pathways are known to induce the production of type I IFNs^38^: the first one is the cytosolic DNA which activates cGAS/STING pathway, as described above, and the second one is the endosomal DNA which is detected by TLR9. Then TLR9 detects endosomal DNA and activates IRF7^29^. In our transcriptomic analysis we highlighted a significant up regulation of *Tlr9* and *Irf7* (Fig. 2). Thus, if the induction of TREX1 expression led to inhibition of the cytosolic DNA pathway, the endosomal pathway could be an alternative for high dose PT to induce the type I interferon pathway.

Moreover, a single fraction of 16.4 Gy proton therapy induced a significant antitumor response in our cancer model, with tumor infiltration of different types of immune cells, such as CD8^+^ T cells, which express granzyme B^+^, translating cytotoxic activity. This is related to the induction of expression of certain chemokines observed in RNA-Seq (such as *Cxcl11*), which contribute to the recruitment of effector T cells in the tumor^30^. CD8^+^ T cell activation is one of the mechanisms involved in the induction of the abscopal effect, which is a rare systemic effect first described more than 60 years ago after conventional radiotherapy^39,40^. Brenneman et al*.*, recently described the first case report of an abscopal effect after proton therapy in a patient with sarcoma^41^. The incidence of this abscopal effect is increased when photon RT is combined with immunotherapy^42^. The association of proton therapy with immunotherapy could therefore yield a beneficial effect both locally and outside of the radiation field in metastatic patients. To compare the percentages of antitumor and protumor immune cells obtained after 16.4 Gy proton and photon X irradiations (as previously published^10^) on our colon carcinoma tumor model, the different panels evaluated were gathered in Supplementary Fig. 2. In antitumor immune cells, no difference was induced, except for NK cells which were enhanced after RT, 7 days post-irradiation, but this difference faded after 14 days. For protumor immune cells, no differences were observed for Mo-MDSC and TAM2 cells. Treg cells maintained their percentages of infiltration cells after PT between 7 and 14 days, in contrary to cells treated with RT that diminished 14 days post-irradiation, but the percentages were very low. As infiltration and immune response of PT remain similar to those presented after RT, the difference of PT efficacy may result from DNA damage and not from immune response. A comprehensive study of cytotoxic activity of lymphocytes T could confirm these findings.

Combinations of immunotherapy with conventional radiotherapy are increasingly being evaluated in preclinical and clinical situations^43,44^. Some studies have attempted to optimize the combination conditions to achieve a radio-induced immune response, leading to an in situ vaccine*,* which may be amplified with immunotherapy by inhibiting tumor microenvironment immunosuppression^45^.

Yet, no published preclinical study has described the effectiveness of combining proton therapy with immunotherapy. We searched for the query string “proton” and “immunotherapy” in the ClinicalTrials.gov database in December 2020, and identified only 4 verified trials worldwide evaluating the safety and/or efficacy of a combination of PT and immunotherapy (NCT03765190; NCT03818776; NCT03267836 and NCT03764787) that are currently ongoing or not yet recruiting. They are all early-stage studies (phase I or II) with small sample sizes (maximum 30 patients). All these trials are investigating proton therapy with anti-PD-1 or anti-PD-L1 safety and efficacy for metastatic cancers (neoplasm or head and neck), non-small cell lung cancer, head and neck cancer or meningioma.

In our experiments, we showed that there was tumor infiltration by Tregs, which have an immunosuppressive effect. These interesting findings could encourage the evaluation of an association of proton therapy with for example an anti-CTLA4 or an anti-CCR4, which, by targeting Tregs, induce reactivation of CD8^+^ T cells against tumor cells^46^. We previously demonstrated with conventional photon treatment that inducing expression of immunotherapy targets by RT could have an impact on the efficacy of the combination of RT with a specific immunotherapy (anti-PD-L1)^10^.

There is a keen interest within the scientific community in developing and evaluating the combination of proton therapy with immunotherapy. Several research teams have expressed an interest, on the assumption that proton therapy could activate the immune response and increase immunotherapy efficacy^20,47–49^. However, to date, no study has demonstrated the concept biologically.

For the first time, we demonstrate here that proton therapy can activate the immune response and can “heat up” the tumor by infiltration of antitumor immune cells. As there is considerable heterogeneity in immune response between different tumor models in the literature, our results obtained on one cell line in a mouse model need to be confirmed using other syngeneic models. Our results pave the way for future studies that could evaluate the effect of the proton therapy dose delivered, and its possible fractionation scheme, the effects of variations in RBE, and the best combination of proton therapy and immunotherapy on different models. It therefore seems essential to evaluate the effect of proton therapy on the expression of specific targets, in order to guide clinicians in the choice of immunotherapy to be combined with proton therapy in future clinical trials.

A single fraction of 16.4 Gy proton therapy led to an interesting induction of immune response biological pathways and immunostimulatory antitumoral effects in an ectopic (subcutaneous) mouse model with a transplanted CT26 colon carcinoma cell line. This study paves the way for future investigations that may explain the improved efficiency of PT via activation of other pathways of immunity response. This study reveals the possible potential of combining proton therapy with immunotherapy in order to enhance tumor control and survival.

## Methods

### Cell culture and animals

The murine colon carcinoma cell line CT26 was purchased from American Type Culture Collection and cultured in RPMI 1640 (Dutscher, France) supplemented with 10% fetal bovine serum (PAN Biotech GmbH, Aidenbach, Germany) at 37 °C in a humidified atmosphere containing 5% CO_2_ and 95% humidity.

**Figure 5:**
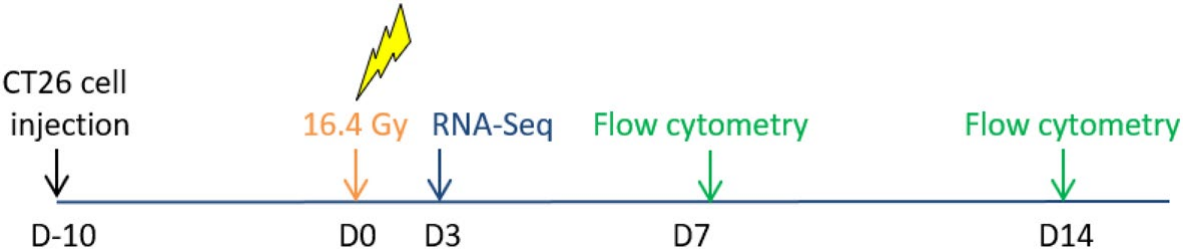
Time scale of the experiments. Ten days after injection of CT26 colon murine cancer cells into Balb/c mice, tumors were irradiated with a single dose of 16.4 Gy compared to a non-irradiated control group. RNA-Seq profiling was performed 3 days post-irradiation and immunomonitoring was performed 7 and 14 days post-irradiation.

Tumor grafting was performed as previously described^10^. Briefly, CT26 cells (5 × 10^5^) were suspended in 100 µl of NaCl and injected subcutaneously in the right flank of immunocompetent 8-week BALB/c female mice (Charles River Laboratories, Saint-Germain-des-Monts, France). Tumor size was measured until tumor volume (TV) reached the limit point of 1500 mm^3^. TV was calculated according to the equation TV = (L × W^2^)/2, where L and W are the length and width of the tumor, respectively. Ten days after injection, mice were randomized to treatment and control groups to obtain an equivalent average tumor volume in each group of 130 ± 20 mm^3^ (n = 5–6 mice for RNA-Seq profiling analysis and n = 4–5 mice for immunomonitoring). Mice were euthanized as soon as the limit point was reached (tumor size ≥ 1500 mm^3^) or 100 days after treatment for the growth delay study; for RNA-Seq experiments, mice were sacrificed 3 days after irradiation; for immunomonitoring experiments mice were sacrificed 7 and 14 days after irradiation (Fig. 5). As requested by the ethics committee and French regulations, the mice were sacrificed by cervical dislocation after general gaze anaesthesia (Isoflurane 2.5%).

All mouse procedures were performed with approved protocols in accordance with the legislation on the use of laboratory animals (directive 2010/63/EU), and with ethical rules for the care and use of animals for research from the small animal ethics committee (C2ea Grand Campus n°105 and C2ea Cremeas n°35, C2ea Icomech n°38) and the French Ministries of Research and Agriculture (APAFIS#13961-2018022215413276 v2, APAFIS#22350-201910091738155 v2 and APAFIS#8235-201612161350414_v1). All procedures used follow ARRIVE guidelines to respect 3R recommendations. Thus, animals are randomized before treatment in order to have comparable tumor volume averages. The numbers of mice in each group allow statistical analysis. The control groups of untreated mice allow the analysis of the effect of the treatment.

### Treatments

Ten days after injection of cancer cells, tumors were irradiated by a single dose of 16.4 Gy under anaesthesia (isoflurane 2.5% mixed with oxygen compact unit, Minerve, France) with a proton beam extracted from CYRCé platform (CYclotron pour la ReCherche et l’Enseignement) in the Institut Pluridisciplinaire Hubert Curien (Strasbourg, France) with an average energy beam of 25 MeV^50^. Using an in-house immobilization bed, tumors were irradiated directly in contact with the collimator with a dose rate of 3.1 Gy/min.

### Flow cytometry

The modulation of the immune response by 16.4 Gy proton therapy was evaluated by flow cytometry at 7 and 14 days after treatment, as previously described^10^. Briefly, after dissection, tumors were dissociated using a mouse tumor dissociation kit (Miltenyi Biotech). To analyze myeloid cell infiltration, tumor cell suspension (10^6^ cells) was stained in Flow Cytometry Staining Buffer (FSB, eBioscience) with specific antibodies according to manufacturer’s recommendation (antibody details are presented in Table S2) for 15 min at room temperature in the dark, washed twice in FSB and analyzed by flow cytometry. To analyze lymphoid cell infiltration, tumor cell suspension was performed according to manufacturer’s recommendation (Miltenyi Biotech). To study CD8^+^ cytotoxicity activity using Granzyme labelling, the tumor cell suspension was cultured on 96-well plates with complete RPMI medium (Dutscher) overnight at 37 °C. During the last 4 h of culture PMA (phorbol 12-myristate 13-acetate; 20 ng/ml; Sigma-Aldrich), ionomycin (1 μg/ml; Sigma-Aldrich), and brefeldin A (2 μl/ml; eBioscience) were added. For lymphoid and myeloid cell infiltration assays, Viability Dye eFluor 780 was used to identify live cells. Flow cytometry acquisition was performed on a Cytoflex 13C cytometer (Beckman Coulter). CytExpert (Beckman Coulter) was used for analysis. For lymphoid and myeloid cell identification and for lymphoid cell functionality, the gating strategies are presented in Supplementary Figure S3.

### RNA extraction, RT-qPCR quantification, RNA sequencing (RNA-Seq) and gene set enrichment analysis

Tumor tissue was dissociated with Minilys tissue homogenizer (Bertin, Ozyme). Then, total RNA was extracted from tumor using Trizol method (Invitrogen).

For RT-qPCR, 1000 ng of RNA was reverse transcribed into cDNA using M-MLV Reverse Transcriptase (10,338,842, Invitrogen) and random primers (10,646,313, Invitrogen). cDNAs were quantified by real-time PCR using a Power SYBR Green Real-time PCR Kit (10,219,284, Fisher Scientific) on a QuantStudio 5 Real Time PCR System (Thermo Fisher Scientific). Relative mRNA levels were determined using the ΔCt method. Values were expressed relative to β- actin. The sequences of the oligonucleotides used are described in Supplementary Table S4.

Single-end transcriptome reads were pseudo-aligned to the UCSC mm 10 reference genome and quantification of gene expressions was performed with the Kallisto algorithm (v 0.44.0)^51^. The program was run with default options.

RNA-Seq profiling analysis was assessed with R software (R version 4.0.3). Differential analysis was performed with DESeq2 R package (version 1.30.0)^52^ using log fold change shrinkage^53^. A gene was considered significantly differentially expressed when the corresponding s-value^54^ was < 0.005 and a log2 fold change ≥ 1. Gene set enrichment analyses were performed using gProfiler2 (v0.2.0)^55^.

### Statistical analysis

The results are expressed as mean ± SEM (standard error of the mean) for immunomonitoring experiments and as mean ± SD (standard deviation) for quantification of genes mRNA relative expression. Figures were designed using GraphPad Prism V8. Software (GraphPad Software, USA). Comparisons between groups were carried out using a non-parametric Mann–Whitney test for immunomonitoring experiments and quantification of genes mRNA relative expression and a Log-rank test for Kaplan Meier curves. Statistical analyses were achieved using SAS version 9.4 (SAS Institute Inc., Cary, NC, USA). A *p*-value < 0.05 was considered statistically significant.

## Supplementary Information

Supplementary Information 1.

Supplementary Information 2.

## References

[1] Rodriguez-Ruiz ME, Vitale I, Harrington KJ, Melero I, Galluzzi L (2020). Immunological impact of cell death signaling driven by radiation on the tumor microenvironment. Nat. Immunol..

[2] Wennerberg E (2017). Immune recognition of irradiated cancer cells. Immunol. Rev..

[3] Friedman EJ (2002). Immune modulation by ionizing radiation and its implications for cancer immunotherapy. Curr. Pharm. Des..

[4] McBride WH (2004). A sense of danger from radiation. Radiat. Res..

[5] Galluzzi L (2020). Consensus guidelines for the definition, detection and interpretation of immunogenic cell death. J. Immunother. Cancer.

[6] Diamond MS (2011). Type I interferon is selectively required by dendritic cells for immune rejection of tumors. J. Exp. Med..

[7] Fuertes MB (2011). Host type I IFN signals are required for antitumor CD8+ T cell responses through CD8{alpha}+ dendritic cells. J. Exp. Med..

[8] Zhang F (2020). Type I interferon response in radiation-induced anti-tumor immunity. Semin. Radiat. Oncol..

[9] Vanpouille-Box C (2017). DNA exonuclease Trex1 regulates radiotherapy-induced tumour immunogenicity. Nat. Commun..

[10] Grapin M (2019). Optimized fractionated radiotherapy with anti-PD-L1 and anti-TIGIT: A promising new combination. J. Immunother. Cancer.

[11] Laurent PA (2020). In-vivo and in-vitro impact of high-dose rate radiotherapy using flattening-filter-free beams on the anti-tumor immune response. Clin. Transl. Radiat. Oncol..

[12] Gameiro SR (2016). Tumor cells surviving exposure to proton or photon radiation share a common immunogenic modulation signature, rendering them more sensitive to T cell-mediated killing. Int. J. Radiat. Oncol. Biol. Phys..

[13] Huang Y (2019). Comparison of the effects of photon, proton and carbon-ion radiation on the ecto-calreticulin exposure in various tumor cell lines. Ann. Transl. Med..

[14] Spina CS (2020). Differential immune modulation with carbon-ion versus photon therapy. Int. J. Radiat. Oncol. Biol. Phys..

[15] Yoshimoto Y (2015). Carbon-ion beams induce production of an immune mediator protein, high mobility group box 1, at levels comparable with X-ray irradiation. J. Radiat. Res..

[16] Fernandez-Gonzalo R, Baatout S, Moreels M (2017). Impact of particle irradiation on the immune system: From the clinic to mars. Front Immunol..

[17] Grau C, Durante M, Georg D, Langendijk JA, Weber DC (2020). Particle therapy in Europe. Mol. Oncol..

[18] Tommasino F, Durante M (2015). Proton radiobiology. Cancers (Basel).

[19] Keisari Y, Kelson I (2021). The potentiation of anti-tumor immunity by tumor abolition with alpha particles, protons, or carbon ion radiation and its enforcement by combination with immunoadjuvants or inhibitors of immune suppressor cells and checkpoint molecules. Cells.

[20] Durante M, Formenti S (2020). Harnessing radiation to improve immunotherapy: Better with particles?. Br. J. Radiol..

[21] R Core Team. R: A Language and Environment for Statistical Computing. *R Foundation for Statistical Computing, Vienna, Austria, URL *https://www.R-project.org (2020).

[22] Durante M (2014). New challenges in high-energy particle radiobiology. Br. J. Radiol..

[23] Ebner DK (2017). The immunoregulatory potential of particle radiation in cancer therapy. Front Immunol..

[24] Durante M, Orecchia R, Loeffler JS (2017). Charged-particle therapy in cancer: Clinical uses and future perspectives. Nat. Rev. Clin. Oncol..

[25] Dunn GP, Koebel CM, Schreiber RD (2006). Interferons, immunity and cancer immunoediting. Nat. Rev. Immunol..

[26] Gajewski TF, Schreiber H, Fu YX (2013). Innate and adaptive immune cells in the tumor microenvironment. Nat. Immunol..

[27] Deng L (2014). STING-dependent cytosolic DNA sensing promotes radiation-induced type I interferon-dependent antitumor immunity in immunogenic tumors. Immunity.

[28] Woo SR (2014). STING-dependent cytosolic DNA sensing mediates innate immune recognition of immunogenic tumors. Immunity.

[29] Kawai T (2004). Interferon-alpha induction through Toll-like receptors involves a direct interaction of IRF7 with MyD88 and TRAF6. Nat. Immunol..

[30] Biswas SK (2006). A distinct and unique transcriptional program expressed by tumor-associated macrophages (defective NF-kappaB and enhanced IRF-3/STAT1 activation). Blood.

[31] Mikucki ME (2015). Non-redundant requirement for CXCR3 signalling during tumoricidal T-cell trafficking across tumour vascular checkpoints. Nat. Commun..

[32] Cai X, Chiu YH, Chen ZJ (2014). The cGAS-cGAMP-STING pathway of cytosolic DNA sensing and signaling. Mol. Cell.

[33] Chen Q, Sun L, Chen ZJ (2016). Regulation and function of the cGAS-STING pathway of cytosolic DNA sensing. Nat. Immunol..

[34] Harding SM (2017). Mitotic progression following DNA damage enables pattern recognition within micronuclei. Nature.

[35] MacDonald KM, Benguerfi S, Harding SM (2020). Alerting the immune system to DNA damage: Micronuclei as mediators. Essays Biochem..

[36] Green LM (2001). Response of thyroid follicular cells to gamma irradiation compared to proton irradiation: I: Initial characterization of DNA damage, micronucleus formation, apoptosis, cell survival, and cell cycle phase redistribution. Radiat. Res..

[37] Luo R (2019). Cisplatin facilitates radiation-induced abscopal effects in conjunction with PD-1 checkpoint blockade through CXCR3/CXCL10-mediated T-cell recruitment. Clin. Cancer Res..

[38] Paludan SR, Reinert LS, Hornung V (2019). DNA-stimulated cell death: Implications for host defence, inflammatory diseases and cancer. Nat. Rev. Immunol..

[39] Demaria S, Formenti SC (2020). The abscopal effect 67 years later: from a side story to center stage. Br. J. Radiol..

[40] Rodriguez-Ruiz ME, Vanpouille-Box C, Melero I, Formenti SC, Demaria S (2018). Immunological mechanisms responsible for radiation-induced abscopal effect. Trends Immunol..

[41] Brenneman RJ (2019). Abscopal effect following proton beam radiotherapy in a patient with inoperable metastatic retroperitoneal sarcoma. Front Oncol..

[42] Ngwa W (2018). Using immunotherapy to boost the abscopal effect. Nat. Rev. Cancer.

[43] Boustani J, Grapin M, Laurent PA, Apetoh L, Mirjolet C (2019). The 6th R of radiobiology: Reactivation of anti-tumor immune response. Cancers (Basel).

[44] Kabiljo J, Laengle J, Bergmann M (2020). From threat to cure: Understanding of virus-induced cell death leads to highly immunogenic oncolytic influenza viruses. Cell Death Discov..

[45] Wennerberg E (2017). Barriers to radiation-induced in situ tumor vaccination. Front Immunol..

[46] Tanaka A, Sakaguchi S (2019). Targeting Treg cells in cancer immunotherapy. Eur. J. Immunol..

[47] Lee HJ, Zeng J, Rengan R (2018). Proton beam therapy and immunotherapy: An emerging partnership for immune activation in non-small cell lung cancer. Transl. Lung Cancer Res..

[48] Kumari S (2020). Immunomodulatory effects of radiotherapy. Int. J. Mol. Sci..

[49] Durante M, Formenti SC (2018). Radiation-induced chromosomal aberrations and immunotherapy: Micronuclei, cytosolic DNA, and interferon-production pathway. Front Oncol..

[50] Constanzo J, Vanstalle M, Finck C, Brasse D, Rousseau M (2019). Dosimetry and characterization of a 25-MeV proton beam line for preclinical radiobiology research. Med. Phys..

[51] Bray NL, Pimentel H, Melsted P, Pachter L (2016). Near-optimal probabilistic RNA-seq quantification. Nat. Biotechnol..

[52] Love MI, Huber W, Anders S (2014). Moderated estimation of fold change and dispersion for RNA-seq data with DESeq2. Genome Biol..

[53] Zhu A, Ibrahim JG, Love MI (2019). Heavy-tailed prior distributions for sequence count data: Removing the noise and preserving large differences. Bioinformatics.

[54] Stephens M (2017). False discovery rates: A new deal. Biostatistics.

[55] Raudvere U (2019). g:Profiler: A web server for functional enrichment analysis and conversions of gene lists (2019 update). Nucl. Acids Res..

