# Supplementary material for: Impact of proton therapy on antitumor immune response
Source: Sci Rep. 2021 Jun 29;11:13444. doi: 10.1038/s41598-021-92942-1 (PMC8241828; doi:10.1038/s41598-021-92942-1)
Supplement: Supplementary file 1 — Supplementary Information 1. [file 41598_2021_92942_MOESM1_ESM.docx]

**Figure S1.** Kaplan Meier survival curves of X-ray irradiation on CT26 tumors volumes implanted on immunocompetent BALB/c mice with 16.4 Gy (red), 3 x 8 Gy (blue), 18 x 2 Gy (green) compared to non-irradiated (NI) tumors (black). n = 9-10 mice per group. Ten days after injection of CT26 colon murine cancer cells into Balb/c mice, tumors were irradiated with a SARRP irradiator (X-Strahl). Complete methods are described in Grapin et al. ^10^.


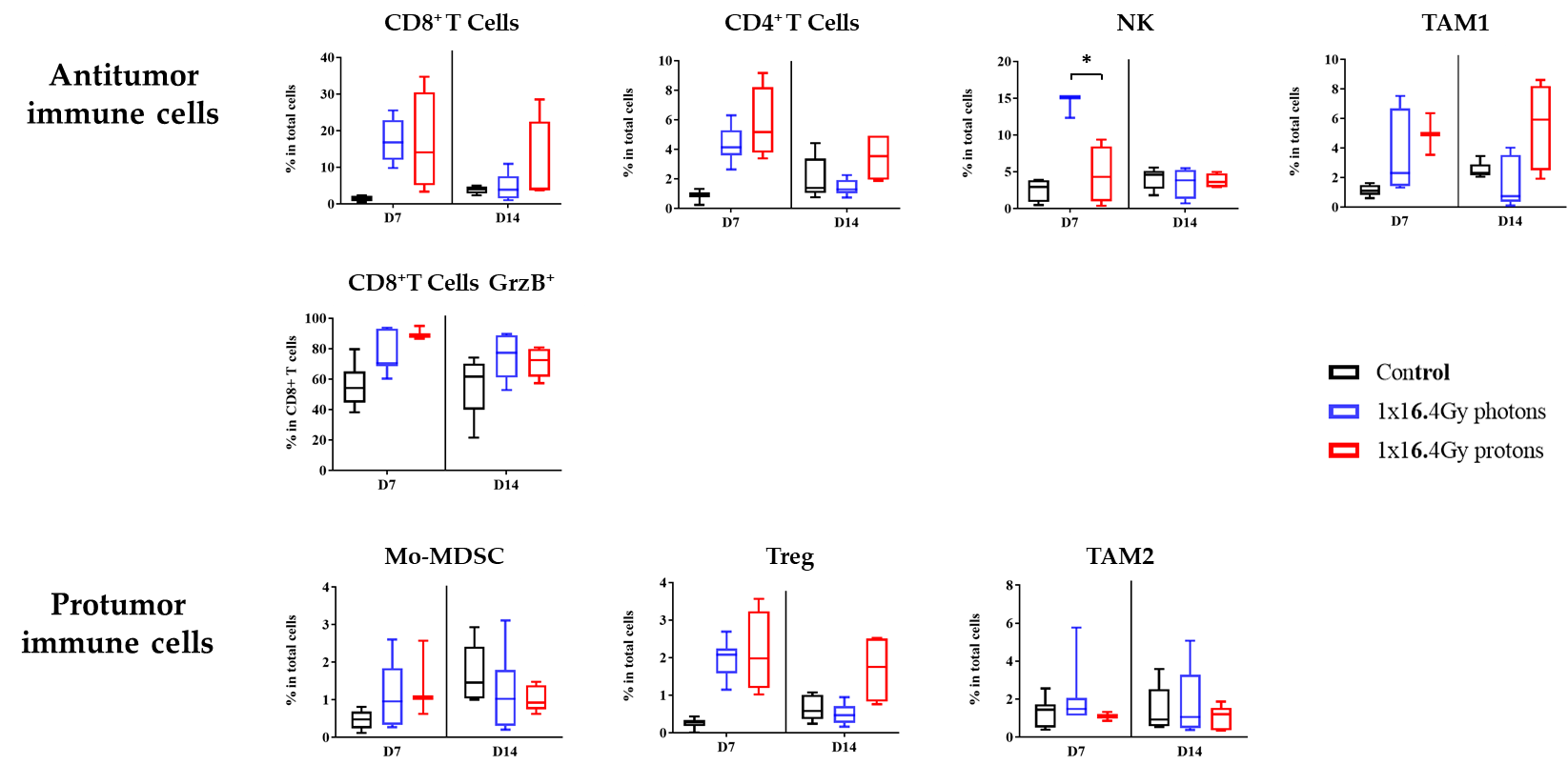


**Figure S2.** Comparison of 16.4 Gy photon (blue) and proton (red) irradiations effects on the modification of the tumor microenvironment. Immunomonitoring of lymphoid and myeloid cells after proton and photon therapies. 7 and 14 days post-irradiation, flow cytometry monitoring (FCM) was performed on dissociated tumors. Antitumor immune cell (CD8+ T cells, CD4+ T cells, Natural Killer (NK) cells, tumor associated macrophages (TAM) 1 and CD8+ granzyme B+ (GrzB) and pro-tumor cell (myeloid derived suppressor cells (Mo-MDSC), Treg T cells and TAM2) infiltration was quantified. All data were expressed in percentages of total cells, except for CD8+T cells Granzym B+ which were expressed in percentages of CD8+T cells. All data are shown with box and whisker plots, with min to max values obtained from 4-6 independent samples per point. The results are expressed as mean ± SEM. The non-parametric Mann-Whitney test was used to compare values obtained after photons vs proton. Excepted for NK tumor infiltration 7 days after treatment (which was significantly higher after photons than after protons), there were no statistically significant differences between photon and proton comparisons. *p<0.05. The photon (X-rays) data are extracted from analysis published in Grapin et al. ^10^.


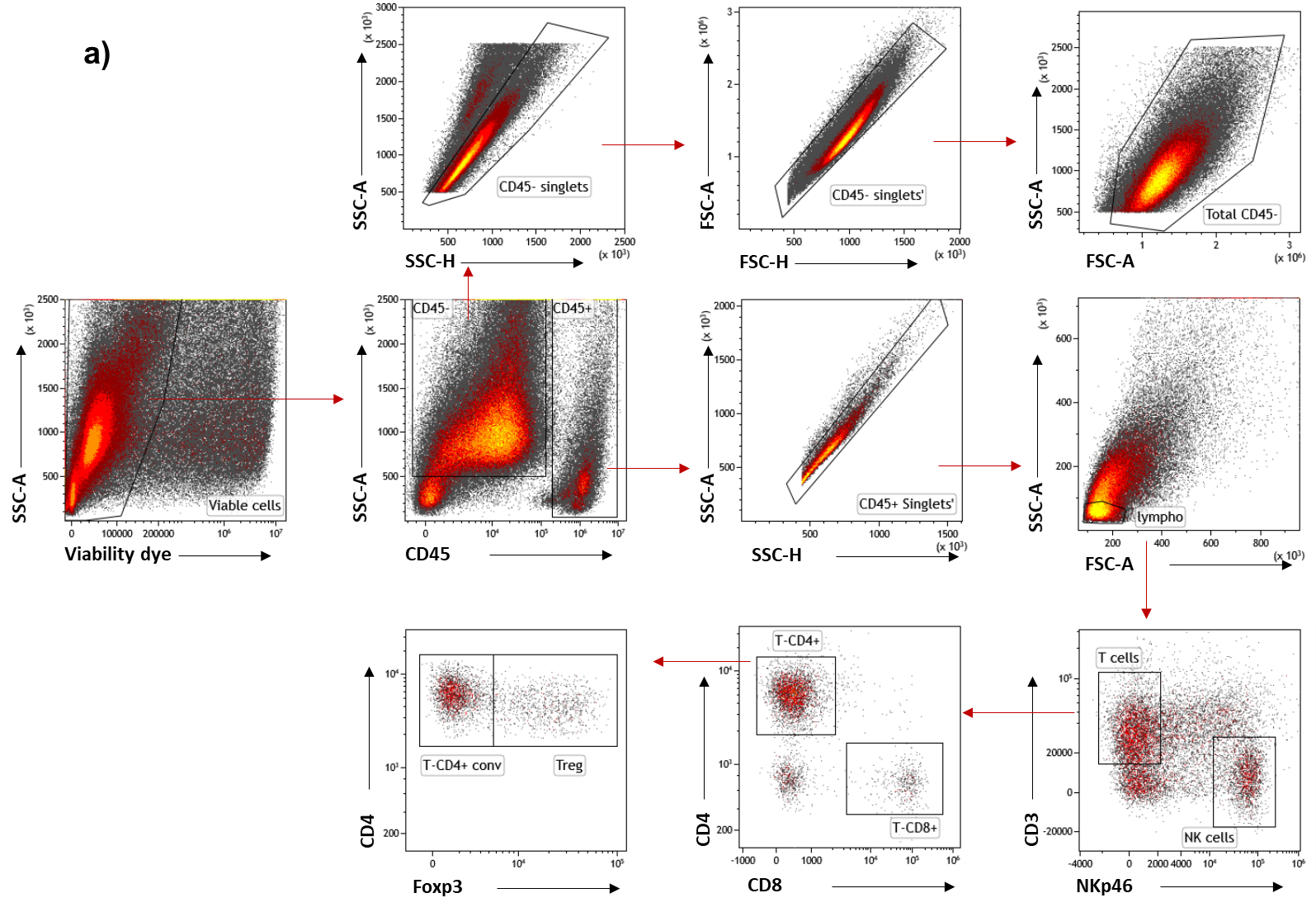


**
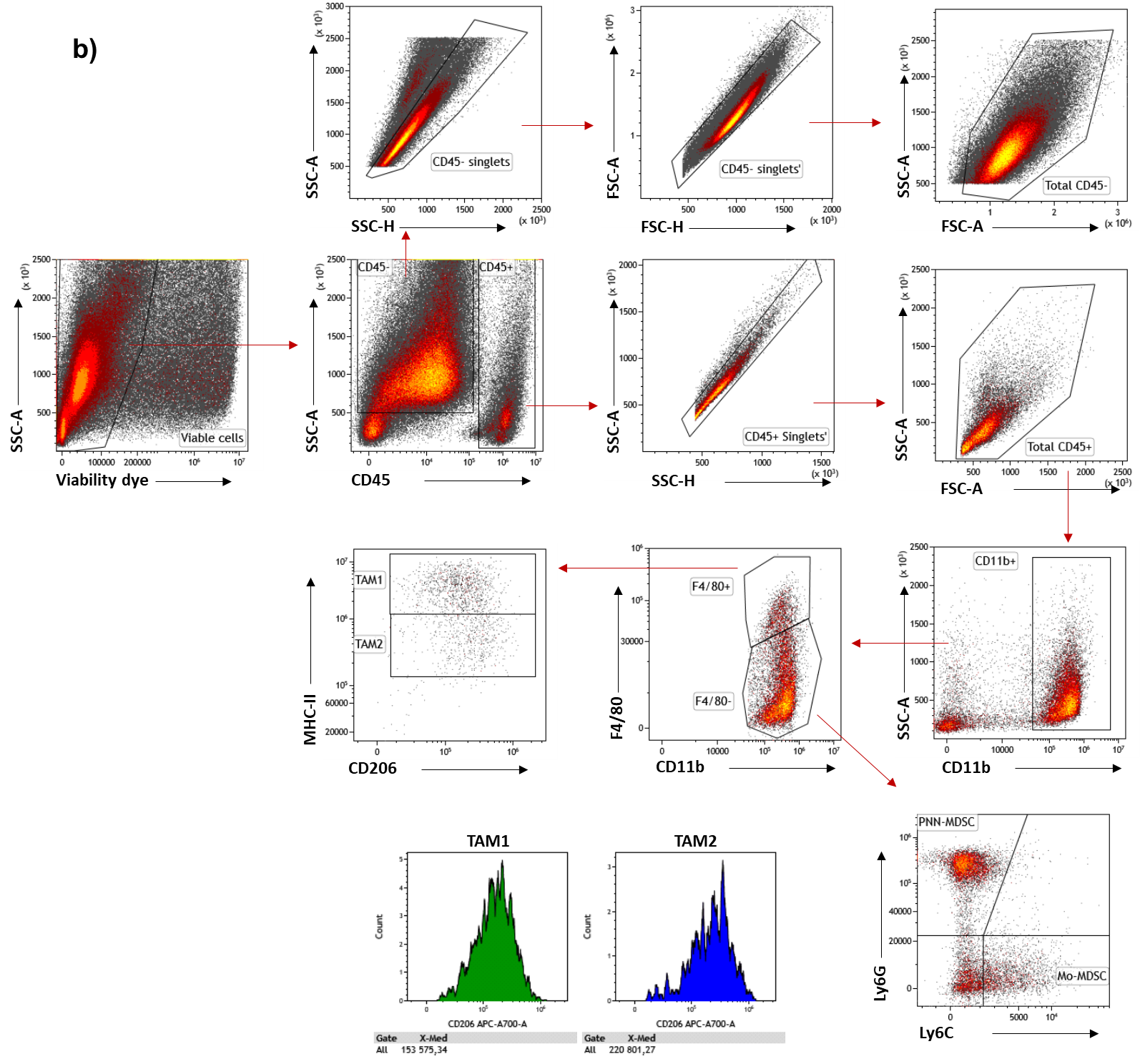
**

**
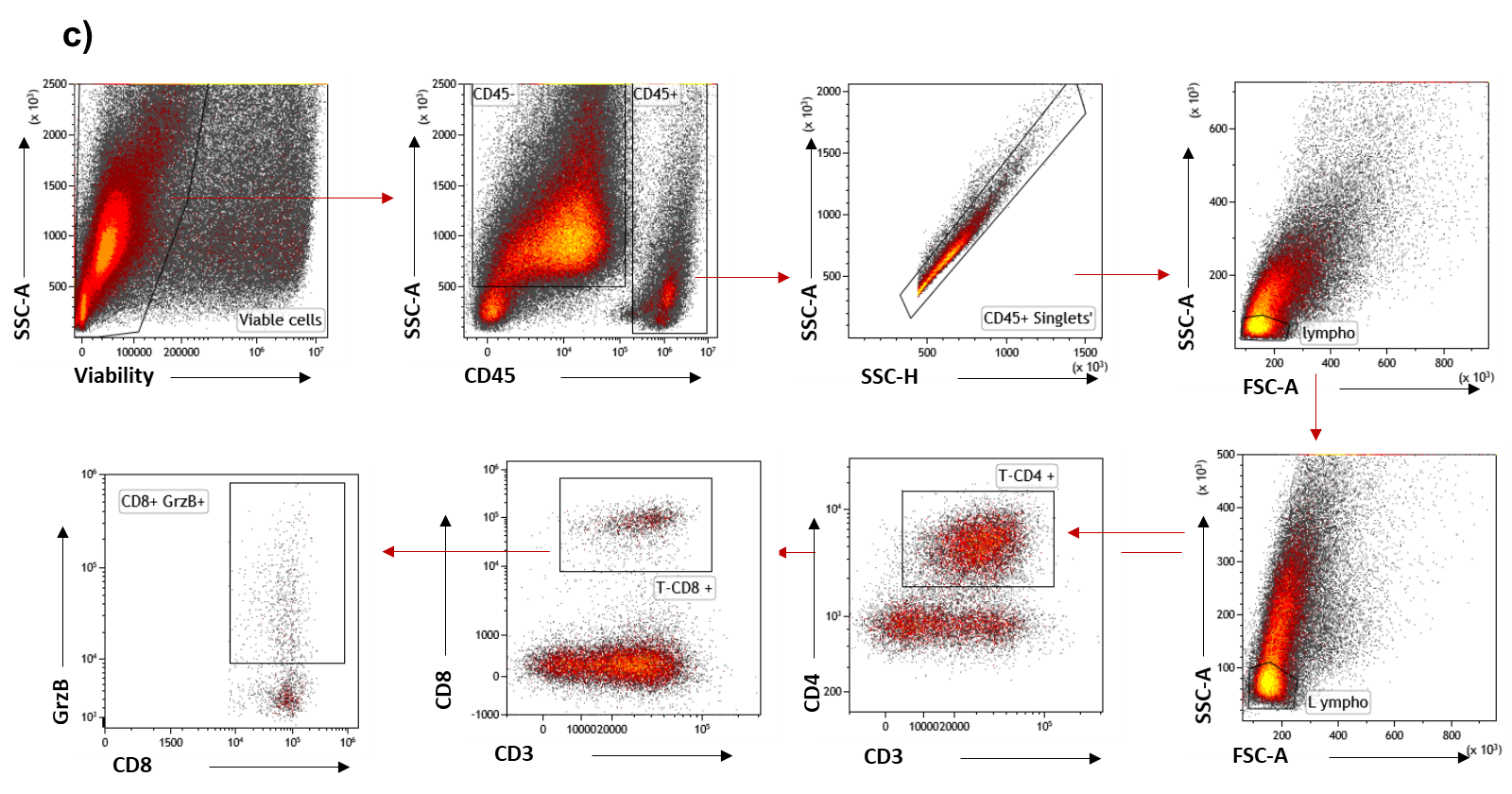
**

**Figure S3.** Gating procedures for flow cytometry. **a)** Gating strategy for lymphoid cells identification and quantification in tumor tissue. After CT26 tumor tissue dissociation, cells were stained with viability dye (eFluor 780), anti-CD45, anti-CD3, anti-CD4, anti-CD8, anti-CD25 and anti-Foxp3 antibodies and analyzed by flow cytometry. The frequency among CD45+ Tils of total T cells (CD45+ CD3+), CD8+ T cells (CD45+ CD3+ CD8+), CD4+ T cells (CD45+ CD3+ CD4+), NK Cells (CD45+CD3-NKp46+), and Treg (CD45+ CD3+ CD4+ Foxp3+) were analyzed. **b)** Gating strategy for myeloid and tumor cells identification and quantification in tumor tissue. After CT26 tumor tissue dissociation, cells were stained with viability dye (eFluor 780), anti-CD45, anti-CD11b, anti-Ly6G, anti-Ly6C, anti-F4/80, anti-MHC-II and anti-CD206 antibodies and analyzed by flow cytometry. The frequency among CD45+ Tils of total myeloid cells (CD45+ CD11b+), TAM2 (CD45+ CD11b+ Ly6G- Ly6Clow F4/80+ MHC-II- CD206high), TAM1 (CD45+ CD11b+ Ly6G- Ly6Clow F4/80+ MHC-II+ CD206+) were analyzed. c) Gating strategy for lymphoid cells functionality quantification in tumor tissue. After CT26 tumor tissue dissociation and stimulation with PMA/ionomycin for 4 hours, cells were stained with viability dye (eFluor 780), anti-CD45, anti-CD3, anti-CD8 and anti-GrzB antibodies and analyzed by flow cytometry. The frequency of GrzB+ CD8+ Tils (CD45+ CD3+ CD8+) were analyzed. CytExpert software v.2.4.0.28 (Beckman Coulter, https://cytexpert.software.informer.com/1.2/) was used for analysis.

**Table S1.** List of the 68 genes differentially expressed between the non-irradiated control and the 16.4 Gy tumors 3 days post-irradiation. s-value < 0.005. Groups contained 5-6 mice.

**Table S2.** List of GO, KEGG and REACTOME categories obtained after enrichment on gProfiler2 after a dose of 16.4 Gy proton therapy on CT26 tumors with an s-value < 0.005 and a Fold Change ≥ 2.

**
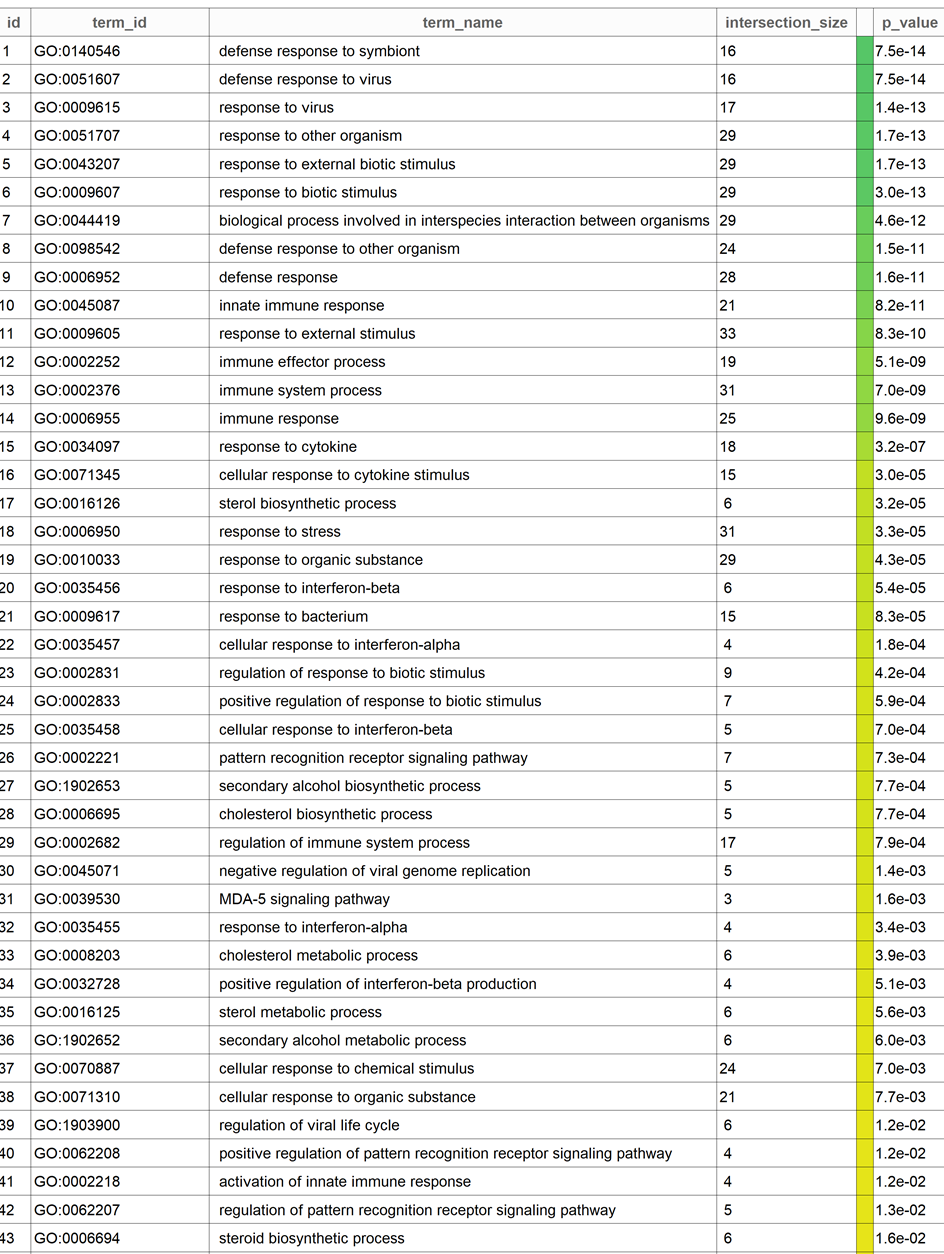
**

**
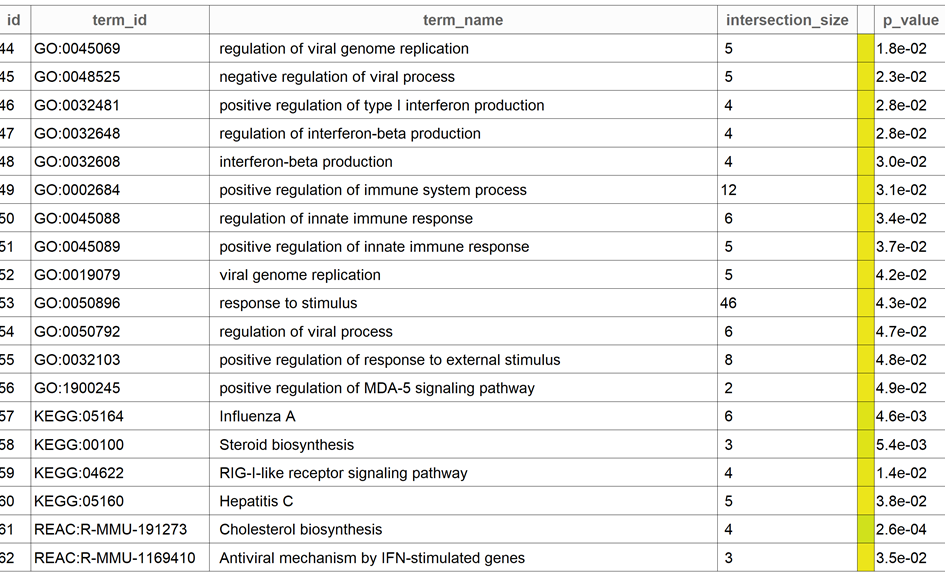
**

**Table S3**. List of antibodies used for identification of myeloid and lymphoid cell and for the study of lymphoid function.

|  | **Marker** | **Fluorochrome** | **Clone** |
| --- | --- | --- | --- |
| **Myeloid panel** | Ly-6C | VioBlue | 1G7.G10 |
|  | CD11b | VioGreen | REA592 |
|  | Ly-6G | Brilliant Violet 785 | 1A8 |
|  | pan-MHC-II | Alexa Fluor 488 | M5/114.15.2 |
|  | CD206 | PE-Dazzle594 | C068C2 |
|  | F4/80 | PerCP-Vio700 | REA126 |
|  | CD45 | APC | REA737 |
|  | Fixable Viability Dye | eFluor 780 | x |
|  |  |  |  |
|  | **Marker** | **Fluorochrome** | **Clone** |
| **Lymphoid panel** | CD4 | VioGreen | REA604 |
|  | CD8 | BV605 | 53-6.7 |
|  | Foxp3 | vio BRIGHT 515 | REA788 |
|  | NKp46 | PE-Vio770 | REA815 |
|  | CD45 | APC | REA737 |
|  | CD3 | A700 | 17A2 |
|  | Fixable Viability Dye | eFluor 780 | - |
|  |  |  |  |
|  | **Marker** | **Fluorochrome** | **Clone** |
| **Function panel** | CD4 | VioGreen | REA604 |
|  | CD8 | BV605 | 53-6.7 |
|  | Granz B | PE | REA226 |
|  | CD45 | APC | REA737 |
|  | CD3ε | Alexa 700 | 17A2 |
|  | Fixable Viability Dye | eFluor 780 | x |

**Table S4.** List of oligonucleotides used for RTqPCR method.

| **Oligonucleotide** | **Supplier** | **Sequence** |
| --- | --- | --- |
| Mouse β*actin* forward (qPCR) | ThermoFisher Scientific | GGCTATGCTCTCCCTCACG |
| Mouse β*actin* reverse (qPCR) |  | CGCTCGGTCAGGATCTTCAT |
| Mouse *cxcl10* forward (qPCR) |  | CCAAGTGCTGCCGTCATTTT |
| Mouse *cxcl10* reverse (qPCR) |  | TTCATCGTGGCAATGATCTCAAC |
| Mouse *trex1* forward (qPCR) | BioRad | Ref : qMmuCED0061616 |
| Mouse *trex1* reverse (qPCR) |  |  |
